# External Application of Ice in Typhus

**Published:** 1853-07

**Authors:** 


*External application of Ice in Typhus.* By Dr. SANDRAS.—The practice consists in the following proceeding:—The abdomen alone is covered with ice, when tympanitis, heat of skin, pain in the abdomen on pressure, and attacks of colic, constitute the chief symptoms. Ice is mixed with linseed-meal, which absorbs the water, and is renewed at frequent intervals in the beginning, when the thawing goes on too quickly from the heat of skin; the application is repeated every quarter of an hour, or even oftener, but less frequently as the heat and fever diminish. First the tympanitis vanishes; then the pain and fever. In hæmorrhages, during typhus, there is no better remedy than ice. Should congestion of the head and stupor supervene, then ice must be applied in a caoutchouc bag to the scalp.—*Med. Times and Gaz.* from *Gaz. des Hop.*, 13, 1853.

